# Intraoperative Patellofemoral Kinematic Acquisition: The Design, Testing, and Validation of a Setup for Clinical Studies

**DOI:** 10.3390/jcm13247784

**Published:** 2024-12-20

**Authors:** Alberto Favaro, Tommaso Bonanzinga, Giulia Avallone, Simone Bignozzi, Marta Costantini, Francesco Iacono

**Affiliations:** 1 IRCCS Humanitas Research Hospital, Via Manzoni 56, Rozzano, 20089 Milan, Italy; tommaso.bonanzinga@hunimed.eu (T.B.); francesco.iacono@humanitas.it (F.I.); 2 Department of Biomedical Sciences, Humanitas University, Via Rita Levi Montalcini 4, Pieve Emanuele, 20072 Milan, Italy; 3 Orthokey Italia S.r.l., Piazza Giacomo Puccini, 26, 50144 Firenze, Italy; g.avallone@orthokey.eu (G.A.); igs.product@orthokey.eu (S.B.); m.costantini@orthokey.eu (M.C.)

**Keywords:** knee, total knee arthroplasty, patellofemoral joint, patellofemoral kinematics, patellofemoral tracking, navigation

## Abstract

**Background/Objectives**: Abnormalities in patellar tracking, often overlooked in surgical planning, have been identified as a contributing factor to total knee arthroplasty (TKA) complications, including anterior knee pain, patellar subluxation, and dislocation. This study aims to evaluate the repeatability of a novel intraoperative setup for assessing patellofemoral kinematics and its interaction with prosthesis design and positioning during surgery. This setup may support personalized alignment techniques in TKA, potentially improving surgical outcomes. **Methods**: Kinematic data were collected under both native and post-TKA conditions, and the Repeatability Coefficient (RC), Intraclass Correlation Coefficient (ICC), and Limits of Agreement of the Mean were calculated to assess measurement reliability. **Results**: RC values indicated high repeatability, with patellar flexion averaging an RC of 1°. Rotation and tilt demonstrated an RC below 1° post-mid-flexion, while patellar shift maintained an RC of approximately 1.6 mm. ICC and the extended Bland and Altman analysis showed an excellent agreement (ICC > 0.9) and an expected mean difference of zero for all the measured parameters. Measurements were consistent across both flexion and extension, and between native and post-TKA conditions. **Conclusions**: The proposed setup for intraoperative patellofemoral kinematic assessment demonstrated high repeatability and practical utility. The approach was found to be non-intrusive to patellar motion tracking and can be robustly integrated into the intraoperative workflow. This method provides a reliable approach for real-time patellar tracking, which may contribute to more personalized and precise TKA procedures, potentially reducing post-surgical dissatisfaction and complications.

## 1. Introduction

While the annual number of total knee arthroplasties (TKAs) continues to increase, the literature indicates that the dissatisfaction rate among unrevised patients remains significant, at approximately 10% [1]. Historically, this dissatisfaction has been attributed to the tibiofemoral joint and the difficulty of restoring the ideal alignment of prosthetic components in a way to ensure proper limb balance, joint stability, and optimal soft tissue tensioning. Consequently, various alignment philosophies have been proposed over time, with recent trends emphasizing patient-specific approaches tailored to individual anatomical and biomechanical characteristics [2,3].

While restoring the natural tibiofemoral joint line is undeniably pivotal, recent studies suggest that a substantial portion of dissatisfaction may be attributed to complications involving the patellofemoral (PF) joint. Issues such as maltracking and instability are frequently implicated, leading to subluxation, dislocation, altered quadriceps function, and anterior knee pain [4,5,6]. These findings highlight the importance of incorporating PF kinematics, as well as its interaction with prosthetic design and positioning, into standard surgical practice. Acquiring PF kinematics pre- and post-TKA would allow us to quantitatively assess how TKA modifies kinematics relative to preoperative conditions. This would facilitate the intraoperative replication of the physiological circular path and the lateral position of the trochlear groove [7,8], ultimately optimizing the anterior compartment and reducing the risk of postoperative complications.

Nonetheless, assessing PF kinematics remains a secondary focus during surgical procedures, primarily due to the lack of easy-to-use, dedicated instrumentation techniques for quantitative evaluation.

Existing solutions for PF tracking, such as imaging-based techniques like MRI [9,10,11] and CT [12,13], face significant limitations. MRI and CT require bulky equipment that is not easily adaptable to surgical procedures, with CT additionally involving exposure to ionizing radiation. Electromagnetic tracking systems, which employ sensors embedded in the bone and tracking via external electromagnetic fields [14], also present challenges, including interference from surrounding metal instruments and the need for close proximity to ensure accurate tracking. These limitations reduce their safety, precision, and practicality in standard surgical workflows.

A promising alternative is optical navigation, which leverages stereophotogrammetric technology to track human bony segments by digitizing anatomical landmarks and using reflective or emitting trackers securely fixed to the bone [15,16,17]. However, current optical systems for PF tracking are scarce, they often lack proper validation with regard to accuracy, and remain confined to experimental contexts. Moreover, the bulky design of trackers [17] can introduce inertial effects that interfere with physiological patellar motion during kinematic acquisition. In other cases [15,16], their permanent fixation to the patella limits practicality in routine surgical procedures.

Additional challenges in PF tracking have emerged in defining a standard coordinate system for the patella due to its unique and irregular morphology. Over the years, various anatomical reference systems have been proposed [18,19,20], differing in their choice of anatomical landmarks and data collection methods. Consequently, results from these studies are highly dependent on dissimilarities in coordinate systems and axis orientations, complicating cross-study comparisons [21].

An ideal solution for PF kinematic evaluation would involve a removable, lightweight, and user-friendly stereophotogrammetric setup capable of intraoperative measurement without interfering with standard surgical instrumentation and procedures. Furthermore, the system should adhere to a recognized, anatomical reference standard, enabling results to be compared across different studies. Such a solution would not only lay the groundwork for future clinical research but also support routine surgical use, where the quantitative assessment of PF kinematics could play a pivotal role in improving patient outcomes.

This paper presents a feasibility study on the design and validation of a novel stereophotogrammetric setup for intraoperative patellar kinematics measurement. The aim is to address all the challenges presented. The proposed setup is assessed for its reliability, repeatability, and practicality in acquiring PF kinematics through rigorous testing involving multiple operators, diverse samples, and various maneuvers.

## 2. Materials and Methods

### 2.1. Experimental Setup

The feasibility of the experimental setup was evaluated using six fresh-frozen hemi-body cadaveric specimens (1 male, 5 females; mean age 87 ± 14.4 years, range 63–101 years; BMI 17.8 ± 4.9). Each specimen included the pelvis and lower limbs, providing a total of twelve leg samples. All knee joints were verified to be free from anatomical defects and a history of prosthetic implant procedures. Additionally, the joint capsules, cruciate and collateral ligaments, and patellar and quadriceps tendons were intact.

To ensure stable fixation during testing, the pelvis was securely mounted on an anatomical table. Metal pins were placed to lock the iliac crests and the groin region in place, achieving reliable immobilization throughout the experimental procedures.

**Figure 1:**
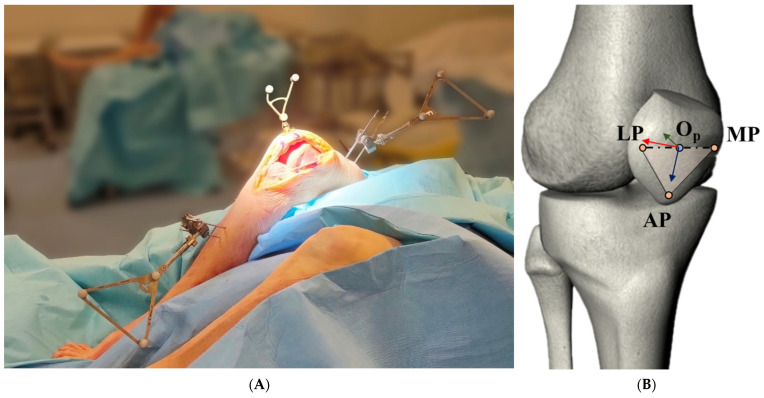
(**A**) Experimental setup: The femoral and tibial trackers are secured with two long pins each, while the patellar tracker is attached to the anterior cortical surface using a single metal screw. All three trackers can be removed by their bases during the TKA procedure. (**B**) Anatomical reference frame of the patella defined from the digitalization of three anatomical points: medial prominence (MP) and lateral prominence (LP), and patellar distal apex (AP). The red, green, and blue arrows represent the Xp, Yp, and Zp axes, respectively.

The intraoperative measurements were conducted using a commercial navigation system (BLU-IGS, software version: 1.4, Orthokey Italia S.r.l., Florence, Italy), which is extensively used in routine clinical practice and research studies. The protocol and accuracy of this system were previously reported in [22,23]. Navigated marker arrays were securely attached to the femur and tibia (Figure 1A). A third, standard pointer-shaped array of markers was used to digitize anatomical landmarks, which were used by the navigation system to define the anatomical reference frame.

In addition to the standard navigation kit, a custom-designed magnetic reference frame was specifically developed for intraoperative PF tracking. This frame was affixed directly to the patella bone via a medial parapatellar approach, following patella exposure. The tracker was securely attached to the patella using a metal screw, designed to minimize the risk of bone damage. Its compact, thin base incorporated a removable frame to ensure it did not interfere with standard surgical procedure. The lightweight structure of the tracker was specifically designed to minimize inertial effects, thereby preserving the natural PF kinematics during the experiments.

### 2.2. Patellar Reference Frame

The anatomical reference system of the patella was defined using a previously recognized and validated approach [20], which involved the use of three key anatomical points on the patella: the medial prominence (MP) and lateral prominence (LP), and the patellar distal apex (AP). The origin of the reference system (Op) was established as the midpoint between MP and LP. The *Y*-axis (Yp) was defined as orthogonal to the plane formed by these points and directed posteriorly. The *Z*-axis (Zp) extended between AP and Op, while the *X*-axis (Xp) was set perpendicular to both Yp and Zp, pointing laterally (Figure 1B). With Xp oriented laterally and Yp posteriorly, the direction of Zp varies, pointing distally or proximally depending on the leg side. According to [19], PF kinematics can be described using four measures: tilt, rotation, flexion, and shift. The first three refer to rotations around Zp, Yp, and Xp, respectively, while shift indicates the patella’s medial–lateral translation along the femoral axis. The Grood and Suntay method [24] widely used for the TF joint was adapted to the PF joint to define these four clinical parameters.

### 2.3. Data Acquisition

For each leg sample, the anatomy was acquired, digitized and displayed on a navigation screen for surgeon convenience. We evaluated PF kinematics during manual flexion and the extension of the leg. The surgeon executed the movements across the full range of motion while monitoring the knee’s condition in real time on the navigation display.

During these maneuvers, the surgeon stood laterally to the sample, positioning their hands under the thigh and calcaneus to prevent interference with the natural kinematics during knee flexion and extension. Kinematic data were recorded under two conditions: first, we captured the native joint kinematics; subsequently, a TKA procedure was performed on the specimen, and the measurements were repeated with prosthetic components in place. To minimize the risk of bone rupture, the patella was not resurfaced during the procedure.

For both the native and post-TKA states, three full flexion–extension repetitions were conducted. During all measurements, the knee joint capsule was sutured to maintain joint stability throughout the experiment.

The navigation system collected data at a frequency of 20 Hz, with kinematic data processed using Modified Akima cubic Hermite interpolation in Matlab (version R2022b, The MathWorks, Inc., Natick, MA, USA). Post-processing uniformly limited all data from the twelve specimens to a 0–120 degree range of flexion–extension. In accordance with [25], measurements were normalized by subtracting the patellar value at full knee extension (zero position).

### 2.4. Statistical Analysis

The statistical analysis was conducted using IBM SPSS version 25.0. We assessed the reliability and repeatability of the method utilizing the Intraclass Correlation Coefficient (ICC), calculated through two-way random effects; absolute agreement; the multiple measurements method [26]; and the Limits of Agreement of the Mean (LOAM), obtained from the extended Bland and Altman plot for multiple repetitions [27].

The Intraclass Correlation Coefficient (ICC) is a reliability index widely used in test–retest, intrarater, and interrater reliability analyses. It is a value between 0 and 1, where values below 0.5 indicate poor reliability, values between 0.5 and 0.75 moderate reliability, values between 0.75 and 0.9 good reliability, and any value above 0.9 indicates excellent reliability.

The Bland–Altman plot is the most common method used to analyze and visualize agreement between repetitions or methods of establishing quantitative outcomes: an expected mean difference different from 0 indicates the presence of bias, i.e., one or more of the repetitions tend to be always greater or smaller than the others, which is not a sign of reliability; and the Limits of Agreement of the Mean (LOAM) allows the expected maximum difference between the repetitions to be quantified, which is useful for understanding the extent of the deviations during the repetitions.

The single-subject Repeatability Coefficient (RC) is calculated for each case as the index used to quantify the value below which the absolute differences between three measurements would lie within a 0.95 probability [28]. The lower the RC, the higher the repeatability of the measure.

## 3. Results

Table 1 presents the RC values for patellar flexion, rotation, tilt, and shift acquired for native joint kinematics across all twelve legs during flexion at four levels: 20°, 40°, 60°, and 80°. These steps were included to represent key stages of knee flexion. Higher RC values are typically observed at 20°, particularly for patellar flexion, due to the patella not fully engaging with the trochlear groove, which acts as a mechanical guide during flexion–extension. Consequently, an unengaged patella is more prone to spurious movement, especially considering the added mass of the attached patella reference frame. At higher knee flexion angles, where the patella engages with the trochlear groove, the RC values tend to decrease, with rotational parameters (patellar flexion, rotation, and tilt) averaging close to 1°. The RC for patellar shift remains approximately 1 mm throughout the movement.

Values related to extension are not detailed here for brevity as they display similar RC outcomes. The same trends apply to post-TKA acquisitions for both flexion and extension movements. A summary of the mean RC values at 80° is provided in Table 2, covering native flexion, native extension, post-TKA flexion, and post-TKA extension.

The ICC and the expected mean difference and LOAM were calculated and are reported in Table 3.

The comparison between the natural PF joint kinematics and post-op kinematics is not the topic of this work, and thus is not discussed.

## 4. Discussion

The present study demonstrated that the proposed setup offers high repeatability and excellent agreement in both flexion–extension and native/post-TKA conditions. The removable patella tracker integrates seamlessly into surgical workflows, enabling precise PF kinematics analysis and making this setup feasible for clinical studies and for optimizing PF tracking in TKA procedures.

### 4.1. Key Findings

The mean repeatability coefficient value of all the subjects is around 1° for PF flexion and below 1° for the PF rotation and tilt after the mid-flexion knee angle is reached. For the shift, the RC is as much as 1.6 mm on average. These results indicate that there is a 95% probability that the rotational error in PF joint evaluation will remain within approximately 1°, with less than a 2 mm error for the patellar shift. Moreover, we performed the ICC and the extended Bland and Altman analysis on the three repetitions of the entire plot from 20° to 80° for the 12 legs. The agreement was excellent (ICC > 0.9) and the expected mean difference among the points of the three plots was zero for all the measured parameters; LOAM values were very small, confirming the previous RC values. These results remain consistent for both flexion and extension, as well as for native and post-TKA conditions, demonstrating that the femoral and patellar TKA components do not interfere with PF motion tracking and that the setup can be robustly used in the operating room for the intraoperative performance of kinematic PF tracking during TKA procedures.

The lightweight, compact patellar tracker is removable and integrates easily into standard computer-assisted TKA workflows. Feedback from surgeons highlighted its user-friendly design and the lack of interference with traditional instruments. This innovative tracker offers a reliable and practical solution for intraoperative PF joint kinematics assessment, providing valuable data without complicating surgical procedures.

### 4.2. Comparison to Existing Solutions

Previous studies [16,17,29] investigated setups for intraoperative PF joint kinematics using commercial navigation systems, but these lacked comprehensive statistical analyses of repeatability and accuracy. While some setups relied on larger, heavier trackers [17] that introduced inertial effects, or non-detachable trackers [15,16] that were impractical for routine intraoperative use, our solution overcomes these limitations. Alternative methods, such as manually tracking an etched point on the patella [29], only provide positional data (x-y-z coordinates) without measuring rotational parameters, limiting their utility for comprehensive kinematic characterization.

### 4.3. Limitations

This study has several limitations. Firstly, the kinematic data were acquired through passive mobilization performed by the surgeon, without muscle activation. While this does not compromise the study’s primary objective of validating the setup, active muscle engagement could produce different kinematic patterns.

Secondly, the joint capsule was sutured during both native and post-TKA kinematic acquisitions to maintain joint stability. Variations in suture tension, excessive tightness or looseness, and loosening over repeated flexion–extension cycles may affect the repeatability of the measurements.

Thirdly, the patella was not resurfaced to prevent bone rupture in the cadaveric specimens. As a result, the repeatability of the setup in resurfaced conditions remains untested. This is an important consideration, as patellar resurfacing is a common practice in TKA and may also influence the stability of the patellar reference frame. Further investigation is needed to address this limitation.

Additionally, the experimental setup was tested exclusively with a single medical team using a medial parapatellar approach. While the results demonstrated high repeatability, broader testing involving multiple surgical teams and varied techniques would be valuable in order to further generalize the findings.

### 4.4. Clinical Implications

The ability to measure PF joint kinematics intraoperatively offers significant potential in terms of improving TKA outcomes by addressing the rotational malalignment of femoral and tibial components. Such measurements could enhance personalized alignment techniques, optimize prosthetic component positioning, and inform prosthesis design—particularly the development of more anatomical trochlear grooves and natural patellar resurfacing.

A key advantage of the proposed setup is its lightweight, easy-to-use, and detachable design, which integrates seamlessly into the surgical workflow without requiring additional instrumentation in the operating room as navigation systems are already routinely employed in TKA procedures. This ensures minimal disruption to standard procedures while providing precise kinematic data, making it a practical and efficient tool for intraoperative use.

Given that intraoperative corrections are far easier to implement than postoperative revisions, a comprehensive understanding of PF joint biomechanics could significantly reduce the incidence and severity of anterior knee pain and other complications following TKA. The proposed setup represents a step forward in bridging the gap between PF biomechanics research and practical surgical applications, contributing to the ongoing refinement of TKA procedures.

## 5. Conclusions

The present study validated a novel setup for intraoperative patellofemoral kinematics, demonstrating repeatability, reliability, and practicality. Its lightweight, removable design integrates easily into standard surgical workflows without interfering with instruments. The setup enables precise patellofemoral kinematics analysis, supporting personalized alignment techniques and improving prosthetic design.

This tool has the potential to significantly improve patient outcomes, reduce postoperative complications, and advance clinical studies and surgical workflows, making it a valuable asset for total knee arthroplasty procedures.

## Figures and Tables

**Table 1:** Repeatability Coefficient (RC) for the patellar flexion, rotation, tilt, and shift measurements.

Leg #	Flexion [°]				Rotation [°]				Tilt [°]				Shift [mm]			
	20°	40°	60°	80°	20°	40°	60°	80°	20°	40°	60°	80°	20°	40°	60°	80°
1	9.7	0.5	0.7	2.6	0.5	0.5	0.2	0.2	0.5	0.5	0.2	0.2	0.7	0.3	0.3	0.7
2	-	2.5	1.0	0.3	-	1.4	0.6	0.0	-	1.6	0.1	0.5	-	1.2	0.9	1.3
3	3.4	5.1	2.1	1.2	0.6	1.2	2.0	1.9	0.8	0.5	0.5	3.3	0.2	1.9	2.8	2.1
4	1.3	0.2	0.6	0.5	0.9	1.0	0.9	0.8	0.1	0.3	0.2	0.1	0.8	0.6	0.4	0.4
5	2.6	1.2	1.3	0.4	1.9	1.8	1.2	0.8	1.4	0.7	0.3	0.5	0.2	1.5	0.7	2.3
6	1.8	2.7	3.0	1.9	0.3	0.6	0.7	0.8	0.3	0.1	0.3	0.2	1.0	1.3	1.3	1.2
7	4.2	3.4	2.4	1.4	0.6	0.1	0.7	0.5	0.8	0.8	0.1	0.2	0.4	0.8	0.5	0.7
8	0.6	1.5	1.2	0.5	0.9	0.3	0.6	0.4	0.5	0.2	0.1	0.1	1.0	0.1	0.0	0.3
9	3.8	4.3	3.5	1.9	0.8	1.0	1.1	1.6	1.0	1.2	0.8	0.7	0.9	1.5	2.2	1.2
10	0.4	0.2	0.9	0.9	0.8	0.3	0.5	0.4	0.8	0.6	0.4	0.2	1.2	0.7	0.7	0.5
11	4.1	2.9	0.5	0.7	1.9	1.7	2.1	2.3	0.7	1.2	0.8	0.8	0.7	1.5	2.6	3.4
12	1.9	1.3	0.6	0.5	0.9	0.8	0.6	0.6	0.8	0.2	0.1	0.1	1.7	0.8	0.7	0.3

**Table 2:** Mean Repeatability Coefficient (RC) at 80° for the patellar flexion, rotation, tilt and shift at the following experimental conditions: native flexion, native extension, post-TKA flexion, and post-TKA extension.

Condition	Movement	Flexion [°]	Rotation [°]	Tilt [°]	Shift [mm]
Native	flexion	1.1	0.9	0.6	1.2
	extension	1.0	0.6	0.5	1.1
Post-TKA	flexion	0.7	1.0	0.7	1.6
	extesion	1.3	0.5	0.5	1.6

**Table 3:** Intraclass Correlation Coefficient (ICC), expected mean difference, and the Limits of Agreement of the Mean (LOAM) obtained from the extended Bland and Altman plot for multiple repetitions performed using the kinematic data.

	ICC (95% CI)	Expected Mean Difference	LOAM
flexion	0.999 (0.999 ÷ 0.999)	0.00 (no bias)	±1.468°
rotation	0.998 (0.998 ÷ 0.999)	0.00 (no bias)	±0.724°
tilt	0.998 (0.998 ÷ 0.998)	0.00 (no bias)	±0.676°
shift	0.991 (0.990 ÷ 0.992)	0.00 (no bias)	±1.280 mm

## Data Availability

The datasets presented in this article are not readily available because the data are part of an ongoing study. Requests to access the datasets should be directed to the corresponding author.
